# MicroRNA-132 regulates recognition memory and synaptic plasticity in the perirhinal cortex

**DOI:** 10.1111/j.1460-9568.2012.08220.x

**Published:** 2012-10

**Authors:** Helen L Scott, Francesco Tamagnini, Katherine E Narduzzo, Joanna L Howarth, Youn-Bok Lee, Liang-Fong Wong, Malcolm W Brown, Elizabeth C Warburton, Zafar I Bashir, James B Uney

**Affiliations:** 1 Henry Wellcome Laboratories for Integrative Neuroscience & Endocrinology & MRC Centre for Synaptic Plasticity, Dorothy Hodgkin Building, University of BristolWhitson Street, Bristol BS1 3NY, UK; 2 School of Physiology and Pharmacology & MRC Centre for Synaptic Plasticity, School of Medical Sciences, University of Bristol, University WalkBristol, UK; 3 University of Bologna, Via ZamboniBologna, Italy

**Keywords:** LTD, memory, miR-132, perirhinal cortex, *Rattus norvegicus*

## Abstract

Evidence suggests that the acquisition of recognition memory depends upon CREB-dependent long-lasting changes in synaptic plasticity in the perirhinal cortex. The CREB-responsive microRNA miR-132 has been shown to regulate synaptic transmission and we set out to investigate a role for this microRNA in recognition memory and its underlying plasticity mechanisms. To this end we mediated the specific overexpression of miR-132 selectively in the rat perirhinal cortex and demonstrated impairment in short-term recognition memory. This functional deficit was associated with a reduction in both long-term depression and long-term potentiation. These results confirm that microRNAs are key coordinators of the intracellular pathways that mediate experience-dependent changes in the brain. In addition, these results demonstrate a role for miR-132 in the neuronal mechanisms underlying the formation of short-term recognition memory.

## Introduction

There is strong evidence that the acquisition and consolidation of the familiarity discrimination component of visual recognition memory is mediated by synaptic plasticity in the perirhinal cortex (PRh). Lesions of PRh produce impairments in the ability of rats and monkeys to distinguish between novel and familiar objects (Zola-Morgan *et al.*, 1989; Meunier *et al.*, 1993; Mumby & Pinel, 1994; Ennaceur *et al.*, 1996). *In vivo* electrophysiological recordings have identified a subset of neurons in the PRh which undergo a long-term decrement of neuronal firing upon presentation of a novel image (Fahy *et al.*, 1993). Further studies have investigated the mechanisms of PRh synaptic plasticity and recognition memory, and have identified many common features revealing that long-term depression (LTD) of synaptic transmission in this region may represent the cellular correlate of visual recognition memory (Massey *et al.*, 2001, 2008; Warburton *et al.*, 2003; Griffiths *et al.*, 2008; Tinsley *et al.*, 2009, 2011a,b). In addition we have revealed the presence of two independent parallel pathways that are involved in the acquisition of recognition memory. Inhibition of kainate receptors or muscarinic acetylcholine receptors (mAChRs) blocks the formation of short-term memories but spares long-term memory, whilst inhibition of NMDA receptors or α7 nicotinic acetylcholine receptors selectively impairs long-term memory (Barker *et al.*, 2006b; Tinsley *et al.*, 2011a,b).

MicroRNAs (miRNAs) are short (22-nucleotide) non-coding RNAs that bind the 3′ untranslated region of target mRNAs to mediate post-transcriptional gene silencing. miRNAs can be activated in response to neuronal activity and therefore offer a highly effective means of controlling the expression of proteins involved in memory formation. One such miRNA is miR-132, the expression of which is regulated by neuronal activity due to the presence of CRE elements in its promoter region (Vo *et al.*, 2005; Nudelman *et al.*, 2010; Wibrand *et al.*, 2010). Several miRNAs, including miR-132, have been shown to regulate the size and number of dendritic spines and alter synaptic activity (Edbauer *et al.*, 2010; Impey *et al.*, 2010; Lambert *et al.*, 2010). miR-132 has been shown to target p250GAP, which regulates Rac1-PAK-mediated dendrospinogenesis (Vo *et al.*, 2005; Wayman *et al.*, 2008; Impey *et al.*, 2010), and methyl CpG-binding protein 2 (MeCP2), a protein involved in dendritic development and synaptogenesis and whose modulation can alter synaptic plasticity (Collins *et al.*, 2004; Asaka *et al.*, 2006; Moretti *et al.*, 2006; Klein *et al.*, 2007). Repression of these target proteins has been shown to account for many of the observed physiological effects of miR-132 (Vo *et al.*, 2005; Klein *et al.*, 2007; Wayman *et al.*, 2008; Impey *et al.*, 2010).

It has recently been reported that a transgenic mouse overexpressing miR-132 throughout the forebrain showed impairments in recognition memory (Hansen *et al.*, 2010). However, no studies have investigated the effect of miR-132 on measures of synaptic plasticity associated with memory formation. To address this question we produced a lentiviral vector to overexpress miR-132 and used this to selectively transduce neurons within the PRh. We then performed both behavioural and electrophysiological analyses to investigate the function of miR-132.

## Materials and methods

### Lentiviral vectors

MiR-132 and its flanking sequence was cloned from rat genomic DNA using the following primers: forward 5′-CTAGCCCCGCAGACACTAGC, reverse 5′-CCCCGCCTCCTCTTGCTCTGTA. The gene was then cloned into a lentiviral plasmid under the control of the CMV promoter and upstream of the enhanced green-fluorescent protein (EGFP) cDNA. miR-3120 was used as a control (Scott *et al.*, 2012). Vesicular stomatitis virus glycoprotein pseudotyped lentivirus was produced by cotransfection of HEK293T cells as described previously (Dull *et al.*, 1998; Wong *et al.*, 2005). Viral titre was calculated by fluorescence activated cell sorting analysis of transduced HEK293T cells.

### Culture of HeLa cells

HeLa cells were maintained in Dulbecco’s modified Eagle’s medium supplemented with foetal bovine serum (10%), penicillin (50 U/mL), streptomycin (50 μg/mL) and l-glutamine (2 mm). Cells were plated in 24-well plates at a density of 40 000 per well and transduced with lentivirus the following day.

### Culture of primary cortical neurons

Cortical neurons were cultured from embryonic day 18 Wistar rat brain as described previously (Howarth *et al.*, 2009). Cortical neurons were transduced with lentivirus after 3 days *in vitro* (DIV).

### Western blotting

Seven days post-transduction cultured neurons were lysed in RIPA buffer [Tris, 50 mm; NaCl, 150 mm; Triton X-100, 1%;sodium deoxycholate, 1%; SDS, 0.1%; and 1× protease inhibitor cocktail (Roche Applied Science), pH 8.0]. Ten micrograms of protein was mixed with an equal volume of sample buffer (Tris, 100 mm; glycerol, 25%; SDS, 2%; bromophenol blue, 0.01%; and DTT, 100 mm; pH 6.8) and run on 8% polyacrylamide gels. Protein was transferred to a PVDF membrane, blocked with 5% milk powder in PBS for 1 hour at room temperature and incubated with primary antibody (1:1000; a kind gift from Tadashi Yamamoto, University of Tokyo) at 4 °C overnight. Membranes were washed in PBS–Tween then incubated with HRP-conjugated secondary antibody (1:10 000; GE Healthcare). Enhanced chemiluminescence was used to visualise the proteins. Membranes were re-probed for α-tubulin (1:2000; Sigma–Aldrich) as a loading control. Densitometry analysis of bands was performed using imagej (NIH) and all measurements normalised to the α-tubulin value. One-way anova tests and *post hoc* Bonferroni’s multiple comparison tests were used to compare expression between groups.

### Transduction of perirhinal cortex

Experiments were performed on 41 adult male Dark Agouti rats (200–250 g; Bantin and Kingman, Hull, UK). Procedures had approval from the University of Bristol ethics committee and were performed in accordance with the United Kingdom Animals Scientific Procedures Act (1986). Transductions were performed under isoflurane-induced anaesthesia as described previously (Griffiths *et al.*, 2008). Equal numbers of viral particles (typically 2 × 10^6^ viral particles per injection) were injected bilaterally in behavioural experiments (36 animals). For electrophysiology experiments miR-312 virus was delivered to one hemisphere whilst EGFP virus was delivered to the contralateral hemisphere to be used as control (five animals). A period of at least 3 weeks was allowed between surgery and the commencement of behavioural testing or electrophysiology to allow recovery and expression of the viral cassette.

### Novel object recognition task

Novel object recognition testing was performed as described previously (Griffiths *et al.*, 2008). Rats were habituated to the arena for 3 days (10 min/day) prior to the start of the novel object recognition task. For the sample phase of the task two identical copies of an object were placed in the corners of the arena and the rat was allowed to freely explore the objects for a maximum of 40 s or until it had been in the arena for 4 min. Following the appropriate delay (20 min or 24 h) the rat was returned to the arena for the test phase (3 min) which involved the presentation of a novel object and a third copy of the object presented during the sample phase. The time spent exploring each of the objects was recorded then the discrimination ratio (D2) was calculated as the difference between the time spent exploring the novel and familiar objects divided by the total time exploring both objects. The use of each object as the novel or familiar object was counter balanced between rats. Each rat completed the task at both delays, with a minimum of 7 days interval. A different set of objects was used for each delay. One-sample *t*-tests were used to determine the significance of the D2 (compared with zero discrimination) for each group. Two-sample *t*-tests were used to determine the significance of the difference in D2 between groups. All tests were two-tailed and used a significance level of *P* < 0.05.

### RNA extraction and RT-PCR

To obtain RNA from the lentiviral-transduced perirhinal cortex rats were killed according to schedule 1 of the United Kingdom Animals Scientific Procedures Act (1986) following the completion of behavioural testing and the PRh was rapidly dissected and transferred to RNA later solution for subsequent RNA extraction. RNA was extracted from PRh and cultured cortical neurons using the miRVANA kit (Ambion). Mature miR-132 levels were measured by quantitative RT-PCR and normalised to the small RNA U6B using miRNA TaqMan Assays (Applied Biosystems). The mean value of three technical repeats was used to calculate the relative expression of miR-132 by the ΔΔ*C*_t_ method for each biological replicate. The mean and SEM values for each condition were then calculated across biological replicates; *n*-numbers in the text refer to biological replicates. One-way anova tests and *post hoc* Bonferroni’s multiple comparison tests were used to determine the significance of viral overexpression in cultured neurons. Two-sample *t*-tests were used to determine the significance of the difference in miR-132 expression between behavioural groups. All tests were two-tailed and used a significance level of *P* < 0.05.

### Northern blotting

Total RNA (2 μg) was mixed with an equal volume of gel loading buffer (Ambion). Samples were denatured for 2 min at 95 °C and separated on a denaturing 15% polyacrylamide (19:1 acrylamide:bis-acrylamide) gel. The gel was then bathed for 5 min in a 0.5 μg/mL solution of ethidium bromide in 1 × TBE. RNA was transferred to an uncharged nylon membrane (Hybond-N; GE Healthcare) by semi-dry technique in 0.5 × TBE at 400 mA. RNA was UV-fixed to membranes at 254–302 nm for 1 min (Geldoc-it; UVP). DNA oligonucleotides, complementary to mature miR-132 (Eurofins MWG) were radio-labelled with γ^32^P (PerkinElmer, USA). Membranes were prehybridised for 1 h at 65 °C. Hybridisation buffer comprised 4 × SSC, 10 × Denhardt’s solution and 0.2% SDS. Membranes were then hybridised with radio labelled probe (15 pm in hybridisation buffer) at 37 °C. After 24 h incubation, membranes were washed three times with constant agitation (4 × SSC with 0.2% SDS). Membranes were then exposed to x-ray film (Amersham Biosciences) for 24 h at room temperature before visualising. Blots were then stripped using 1 × SSC and 0.1% SDS for 30 min at 60 °C before membranes were re-probed for total RNA.

### Histology

Following the completion of behavioural testing, rats were terminally anaesthetised with pentobarbitone and transcardially perfused with PFA (4% in PBS). Brains were post-fixed in PFA for at least 48 hours before cryoprotection (40% sucrose in PBS). 30 μm sections were cut using a cryostat and sections stored at −20 °C in cryoprotection solution (30% sucrose, 1% PVP-40 and 30% ethylene glycol in PBS). Sections were mounted onto slides and examined for EGFP fluorescence using a Leica DMRB microscope. Images were captured using a Leica DFC340FX camera.

### Electrophysiology

Following killing with isoflurane, brains were rapidly removed and placed in ice-cold artificial cerebrospinal fluid (aCSF) oxygenated with 95% O_2_ and 5% CO_2_. ACSF comprised (in mm) NaCl, 124; KCl, 3; NaHCO_3_, 26; NaH_2_PO_4_, 1.25; CaCl_2_, 2; MgSO_4_, 1; and d-glucose, 10. Horizontal slices (400 μm) that included hippocampus, PRh, entorhinal and temporal cortices were cut using a vibroslice (WPI, UK). All slices were left to recover in oxygenated aCSF at room temperature for at least 1 h prior to recording. After recovery, a single slice was placed in a submerged recording chamber and superfused with oxygenated aCSF (temperature, 30–32 °C; flow rate, 2 mL/min).

Evoked field excitatory postsynaptic potentials (fEPSPs) were recorded from layers II and III of PRh. Stimulating electrodes were placed on both caudal and rostral sides 0.5 mm from the recording electrode. Stimuli (constant voltage) were delivered alternately to the two stimulating electrodes (each electrode 0.033 Hz). Input–output curves were produced by stimulating initially at 0 V and then at increasing intensities in 10 V steps until a maximal fEPSP was achieved. The minimum intensity to produce an fEPSP discernible from the noise across experiments ranged between 5 and 7V. fEPSPs were reduced to 50–60% of maximum amplitude to achieve a baseline of synaptic transmission before application of stimulating protocols. After a baseline of at least 30 min, high-frequency stimulation (HFS; four trains, each of 100 Hz, 1 s, every 15 s) was delivered on the caudal input to induce long-term potentiation (LTP). Subsequently, 1 hour post-tetanus, carbachol (CCh; 20 μm) was bath-applied for 10 min and the recording continued for a further hour. Bath application of CCh influenced both caudal and rostral input-evoked fEPSPs without significant differences between the two inputs within the same slice (i.e. the previous HFS delivered to the caudal input did not affect the induction of CCh-LTD). fEPSPs were recorded and analysed on-line with winltp software (Anderson & Collingridge, 2007). The peak amplitude of evoked fEPSPs was measured and expressed as percentage of the baseline. For CCh experiments fEPSPs were renormalized to the new baseline prior to addition of CCh. There was no difference between the effects of CCh on fEPSPs recorded from the two inputs and therefore they were averaged. Data pooled across slices are expressed as means ± SEM. LTP and CCh-LTD were measured at 60 and 50 min post-induction respectively. Comparisons of LTP and LTD between EGFP- and miR-132-transduced slices were made over the last data point (mean response over 2 min) for each experiment. The significance of plasticity changes (LTP and CCh-LTD) was established using either paired or unpaired two-tailed *t*-tests as appropriate. Only one slice was used from each transduced hemisphere.

## Results

**Fig 1:**
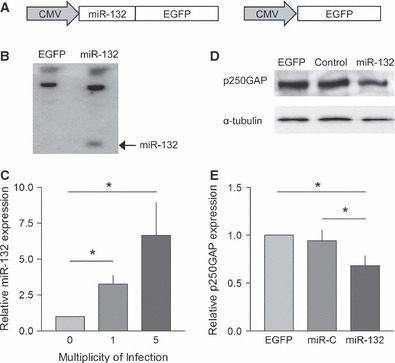
Lentiviral-mediated overexpression of miR-132. (A) The miR-132 gene was cloned downstream of a CMV promoter before the EGFP coding sequence. The pre-miRNA sequence is excised from the mRNA, allowing expression of both miR-132 and EGPF from a single transcript. (B) Northern blot showing overexpression of miR-132 following transduction with the miR-132 lentivirus. (C) TaqMan quantification of miR-132 showing increasing expression of the mature miRNA when primary neurons are transduced with miR-132 lentivirus at increasing multiplicity of infection (*P < 0.05; *n* = 4 independent cultures). (D) Example Western blot showing relative expression of p250GAP and α-tubulin. (E) Summary of Western blot data; overexpression of miR-132 reduced the expression of p250GAP (*P < 0.05; *n* = 6).

The miR-132 gene was cloned from rat genomic DNA and inserted upstream of the EGFP coding sequence in the lentiviral cassette (Fig. 1A). To validate the virus, HeLa cells were transduced and EGFP fluorescence was examined to confirm transduction. RNA was extracted and subjected to Northern blot analysis to confirm expression of the mature miRNA (Fig. 1B). Primary cortical neurons transduced with this vector demonstrated EGFP fluorescence along with increased levels of miR-132 compared to control neurons as measured by TaqMan assay (*F*_2,9_ = 4.392, *P* < 0.05; Fig. 1C).To further validate the virus we confirmed knockdown of the miR-132 target p250GAP. Primary cortical neurons were transduced with either the miR-132 virus, an EGFP-only virus or virus expressing a control miRNA, and protein lysates were collected 7 days post-transduction. Western blotting showed reduced levels of p250GAP in the miR-132-transduced neurons (*F*_2,20_ = 6.136, *P* < 0.05, *n* = 6; Fig. 1D and E) compared to either the EGFP or miRNA control, confirming that the miR-132 expressed from the viral cassette was processed to a functioning mature miRNA. As there was no effect of the control miRNA compared to EGFP the EGFP vector was used a control in the subsequent experiments.

**Fig 2:**
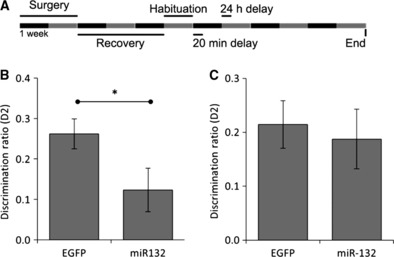
miR-132 impaired short-term recognition memory in the perirhinal cortex. (A) Schematic representation of the time course of behavioural experiments. (B) Rats with bilateral miR-132 transductions of the PRh showed a significant impairment in the novel object recognition paradigm task performed with a 20-min delay when compared to EGFP controls (*P < 0.05; EGFP *n* = 18; miR-132 *n* = 18). (C) There was no impairment in novel object recognition paradigm at a 24-h delay when the PRh was transduced with miR-132 (*P* = 0.704; EGFP *n* = 18; miR-132 *n* = 18). All *n*-numbers refer to the number of animals in each group.

To investigate function *in vivo* we injected lentiviral miR-132-expressing vectors bilaterally into the PRh and assessed recognition memory using the novel object recognition paradigm (Fig 2A). We observed that short-term memory (20-min delay) was significantly impaired in miR-132-transduced rats compared to EGFP controls (miR-132 D2, 0.123 ± 0.052, *n* = 18; EGFP D2, 0.262 ± 0.036, *n* = 18; *P* < 0.05; Fig. 2B). Long-term memory (24-h delay) was not affected by miR-132 overexpression (miR-132 D2, 0.187 ± 0.054, *n* = 18; EGFP D2, 0.214 ± 0.043, *n* = 18; *P* = 0.704; Fig 2C). There was no bias for any of the objects used as shown by the exploration times for each object during the sample phase (Table 1). Similarly there were no differences in total exploration time during each phase between groups (Table 2).

**Table 1:** Exploration times of individual objects during the sample phase

Exploration times (s; mean ± SEM)			
Short-term memory (20 min delay)		Long-term memory (24 h delay)	
	
Object A	Object B	Object A	Object B
10.824 ± 1.079	10.308 ± 0.857	7.115 ± 1.107	9.990 ± 1.161

Presented are the mean exploration times of each object during the sample phase at each delay. There were no significant differences between the exploration times.

**Table 2:** Exploration times during sample and test phases at each delay

Exploration times (s; mean ± SEM)				

Short-term memory (20 min delay)			Long-term memory (24 h delay)	
	
Virus	Sample	Test	Sample	Test
EGFP	23.847 ± 1.627	18.037 ± 1.317	18.935 ± 1.068	19.042 ± 1.476
miR-132	23.463 ± 1.989	17.777 ± 1.320	17.469 ± 1.015	19.279 ± 0.970

Presented are the mean exploration times during the sample and test phases for each group of rats at each delay. There were no significant differences between the EGFP- and miR-132-transduced rats.

**Fig 3:**
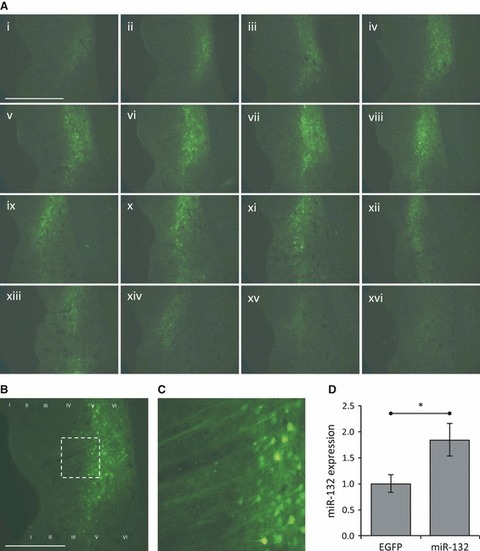
Overexpression of miR-132 in the perirhinal cortex. (A) EGFP expression following stereotaxic injection of miR-132 virus into the perirhinal cortex. 30 μm coronal sections were made through the region containing PRh (every third section is shown). EGFP expressing transduced neurons were identified from bregma −4.8 to −6.3 mm. The centre of the injection site appears level with the rhinal sulcus and it extends up to 1 mm in either direction. (B, C) Magnifications of image (A vi), showing that primarily neuronal cells were transduced. (D) TaqMan quantification of miR-132 expression in the PRh of a subset of the rats used in Fig. 2. There was a significant increase in miR-132 expression in miR-132-transduced PRh compared to EGFP-transduced PRh (*P < 0.05; EGFP, *n* = 8 hemispheres; miR-132, *n* = 8 hemispheres). Scale bar, 1 mm.

To confirm viral transduction of PRh a subset of rats were perfused 2 weeks after the behavioural testing and slices of the brain examined for EGFP fluorescence. We observed transduction of a large region of PRh particularly in the deeper layers. The transduced neurons were seen over an anterior–posterior range of > 1 mm within the region from bregma −4.8 to −6.3 mm (Fig. 3A–C). To quantify the expression of miR-132 in transduced PRh we extracted the RNA from this region of a second group of rats 2 weeks after behavioural testing and analysed using a miR-132-specific TaqMan assay. We observed a nearly twofold increase in miR-132 levels in RNA from miR-132-transduced PRh compared to EGFP-transduced control (miR-132, 1.84 ± 0.314, *n* = 8 hemispheres; EGFP, 1.00 ± 0.168, *n* = 8 hemispheres; *P* < 0.05; Fig. 3D).

**Fig 4:**
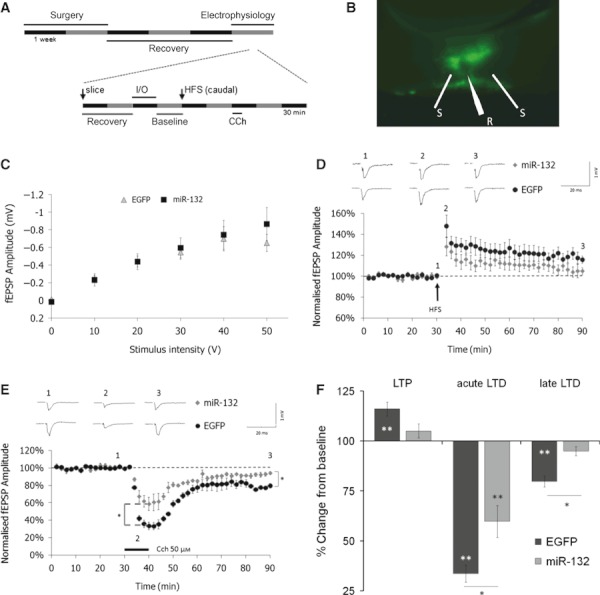
Overexpression of miR-132 reduced plasticity in the PRh. (A) Schematic representation of the experimental design. (B) Image of transduced PRh slice, showing position of stimulating (S) and recording (R) electrodes. (C) Plot of fEPSP amplitude against stimulus intensity showing no difference in the input–output relationship in PRh slices transduced with EGFP (*n* = 5) and miR-132 (*n* = 5). Points represent mean ± SEM in this and all subsequent plots. (D) HFS (black arrow) induced LTP in EGFP-transduced PRh (*n* = 5; *P < 0.01) but not in miR-132-transduced PRh (*n* = 5; *P* = 0.102). Each *n*-number represents the number of slices; only one slice was used from each transduced hemisphere. (E) Normalised fEPSP amplitude against time demonstrating that CCh 20 μm (black bar) induced strong acute depression followed by LTD in EGFP-transduced PRh (*n* = 3). In miR-132-transduced PRh the acute depression induced by CCh was significantly reduced compared to control EGFP-transduced PRh (*P < 0.05) and the late phase of LTD was also impaired (*P < 0.05). (F) Summary of plasticity experiments: HFS failed to induce LTP in miR-132-transduced PRh although there was no significant difference between the groups. There was a significant difference in fEPSP amplitude between EGFP- and miR-132-transduced PRh in both the acute and late phases of CCH-LTD.*P < 0.05, **P < 0.01; asterisks within bars indicate a significant difference from the baseline and those above or below indicate significant differences between groups.

We next investigated the cellular mechanisms underlying the miR-132-induced memory impairment. To achieve this, miR-132 was injected into the left PRh and EGFP injected into the right PRh of each animal. We then performed electrophysiological recordings in acute slices of PRh following these *in vivo* viral transductions (only one slice was used from each transduced hemisphere; Fig. 4A and B). Transduction of PRh with miR-132 lentivirus did not affect basal synaptic activity as shown by input–output analysis (Fig. 4C).

After the recording of a stable baseline (30 min) HFS was delivered to the caudal input whilst the rostral input served as a control (for clarity, data for the rostral input are not shown). HFS induced LTP of the fEPSP in slices from the EGFP-transduced hemisphere (60 min post-HFS, 116.0 ± 3.5% of baseline, *n* = 5 slices; *P* < 0.01; Fig. 4D and F). Slices from the miR-132-transduced hemisphere showed no significant LTP (60 min post-HFS, 105.0 ± 3.5% of baseline, *n* = 5 slices; *P* = 0.102; Fig. 4D and F). However, the fEPSP amplitude one hour following HFS did not significantly differ between EGFP- and miR-132-transduced slices (Fig 4F; *P* = 0.058).

Subsequently, we investigated whether overexpression of miR-132 was interfering with mAChR-dependent plasticity mechanisms, as we have shown previously (Warburton *et al.*, 2003; Tinsley *et al.*, 2011a,b) that blockade of mAChRs by scopolamine produces similar impairments in recognition memory as the overexpression of miR-132 observed in the current study (i.e. impairment at short delay but not at long delay). Application of CCh to acute PRh slices induces a long-lasting depression of synaptic transmission (Massey *et al.*, 2001). In acute slices from the EGFP-transduced hemisphere, application of CCh (20 μm) induced a large acute depression of synaptic transmission measured immediately before CCh washout (40 min fEPSP, 33.6 ± 4.3% of baseline, *n* = 3 slices, *P* < 0.01; Fig. 4E and F). In slices from the miR-132-transduced hemisphere there was also an acute depression of synaptic transmission (40 min fEPSP, 59.7 ± 8.0% of baseline, *n* = 3 slices, *P* < 0.01; Fig. 4E and F) but this was significantly reduced compared with the depression in control EGFP-transduced slices (Fig 4F; *P* < 0.05). The acute depression was followed by long-lasting depression in slices from the EGFP-transduced hemisphere (50 min post-CCh, 79.7 ± 2.7% of baseline, *n* = 3 slices, *P* < 0.01; Fig. 4E and F). However, in slices from the miR-132-transduced hemisphere there was no long-term depression and the responses returned to baseline amplitude (50 min post-CCh, 94.9 ± 2.3% of baseline, *n* = 3 slices, *P* = 0.35; Fig. 4E and F).The fEPSP amplitude 50 min after the washout of CCh was significantly smaller in the EGFP-transduced slices than in the miR-132-transduced slices (Fig 4F; *P* < 0.05).

## Discussion

There is increasing evidence that miRNAs play a pivotal role in regulating synaptic function. We have now shown that the specific expression of miR-132 within the PRh impairs short-term recognition memory, a process known to be dependent on the activity of the PRh. Furthermore we have shown, in miR-132-transduced PRh slices, deficits in synaptic plasticity that are probably substrates for the behavioural impairment.

Hansen *et al.* (2010) demonstrated impaired recognition memory in transgenic mice overexpressing miR-132 throughout the brain. Hence, the overexpression of miR-132 could potentially mediate its effects during neuronal development and by altering gene expression in multiple brain regions. Our results confirm and refine this previous study by showing that overexpression of miR-132 that is restricted to the adult PRh leads to impairment in recognition memory, and also highlight changes in synaptic plasticity that may underlie this impairment. It is interesting to note that only short-term memory mechanisms were affected by the overexpression of miR-132. We have previously demonstrated dissociation between the pathways that underlie short- and long-term recognition memory. Inhibition of either kainite receptors or mAChRs impairs only short-term memory leaving long-term memory intact (Warburton *et al.*, 2003; Barker *et al.*, 2006a,b; Tinsley *et al.*, 2011a,b). In contrast, inhibition of NMDA receptors, group I and group II metabotropic glutamate receptors, nicotinic acetylcholine receptors or voltage-dependent calcium channels spares short-term memory but abolishes long-term memory (Barker *et al.*, 2006a,b; Seoane *et al.*, 2009; Tinsley *et al.*, 2011a,b). These findings clearly demonstrate the presence of at least two independent pathways for the acquisition of recognition memory, with different temporal properties of induction and duration. Our data suggest that miR-132 downregulates proteins whose expression is required for the fast-onset but short-duration memory pathways. Interestingly, activation of many of the receptors and channels required for the slow-onset long-lasting mechanisms would result in an increase in intracellular calcium concentration, which could lead to the phosphorylation of CREB (which we have previously demonstrated to be required for long-term memory; Warburton *et al.*, 2005) and the transcription of miR-132. Therefore a delayed, downstream, increase in miR-132 may actually be permissive to long-term memory.

miR-132 targets p250GAP, a Rac1 GTPase-activating protein (Vo *et al.*, 2005). By repressing p250GAP levels miR-132 increases (via Rac1–PAK signalling) the activity of the actin depolymerising protein *n*-cofilin (Impey *et al.*, 2010). This modulation of actin turnover in dendritic spines has been shown to regulate both spine size and spine density (Edbauer *et al.*, 2010; Hansen *et al.*, 2010; Impey *et al.*, 2010). Inhibition of miR-132 in primary hippocampal cultures decreased spine density and size (Impey *et al.*, 2010) whereas overexpression *in vivo* increased spine density (Hansen *et al.*, 2010). These morphological effects are accompanied by changes in basal synaptic transmission. Although there is some variability between individual studies, miR-132 generally appears to have a positive effect on both the frequency and amplitude of mEPSCs (Edbauer *et al.*, 2010; Impey *et al.*, 2010; Lambert *et al.*, 2010) and also modulates short-term synaptic plasticity (Lambert *et al.*, 2010).

We now demonstrate that miR-132 can also regulate longer-term synaptic plasticity. Application of the mAChR agonist carbachol to slices of PRh results is a large acute depression of synaptic transmission (Massey *et al.*, 2001). In slices of PRh that had been transduced *in vivo* with miR-132 this acute depression was significantly reduced. The time-course of the acute phase of CCh-LTD coincides with the period at which a behavioural deficit was observed in the miR-132-overexpressing rats and, furthermore, we have previously reported that activity of mAChRs is required for novel object recognition memory at this time delay (Warburton *et al.*, 2003; Tinsley *et al.*, 2011a,b). The reduced acute depression and impairment of novel object recognition reported in this paper suggests an impairment in mAChR signalling caused by the overexpression of miR-132. The targets of miR-132 responsible for this deficit are yet to be elucidated.

The effects of miR-132 overexpression of the late phase of CCh LTD and on LTP were more modest. Both these forms of plasticity were impaired in slices from miR-132-transduced slices compared to controls although the effects were small compared to the acute CCh-LTD deficit. We did not observe any effect of miR-132 overexpression on long-term memory tested at 24 h so one would not necessarily predict effects on the later phases of plasticity. The impairments in both LTD and LTP indicate that this miRNA has a general negative effect on synaptic plasticity mechanisms and does not shift the balance towards either depression or potentiation. The impairment in LTD would be predicted by the overexpression of miR-132 reducing the actin depolymerisation underlying LTD-associated spine shrinkage (Zhou *et al.*, 2004). However, one would predict the opposite effect on LTP (Fortin *et al.*, 2010). It is possible that the impairment we find in LTP is due to some homeostatic adjustment to the chronic overexpression of miR-132, but the exact mechanisms remain to be elucidated. Tognini & Pizzorusso (2012) have proposed a model whereby precise control of miR-132 levels is required for regulation of synaptic plasticity. They suggest that if levels are too high then dendritic spines will be excessively stable, impairing plasticity; likewise, if levels are too low then the spines will be very unstable, also hindering plasticity.

Whilst this manuscript was in preparation two groups have demonstrated that miR-132 regulates experience-dependent ocular dominance plasticity (Mellios *et al.*, 2011; Tognini *et al.*, 2011). Visual activity was shown to increase the expression of miR-132 in primary visual cortex. Expression of a sponge construct to inhibit the function of miR-132 blocked ocular dominance plasticity induced by monocular deprivation in control animals (Mellios *et al.*, 2011; Tognini *et al.*, 2011).

Our observations give empirical support to the hypothesis that miR-132 regulates experience-dependent synaptic function. We demonstrate that the impaired plasticity seen as a result of miR-132 overexpression underlies a functional deficit in short-term recognition memory. These results support a role for miRNAs in co-ordinating the complex changes in protein expression that underpin the synaptic mechanisms that control memory acquisition.

These data provide novel insight into the importance of miR-132 in regulating the neuronal function that underpins learning and memory.

## Abbreviations

CCh: carbachol
D2: discrimination ratio
DIV: days *in vitro*
EGFP: enhanced green-fluorescent protein
fEPSP: field excitatory postsynaptic potential
HFS: high-frequency stimulation
LTD: long-term depression
LTP: long-term potentiation
mAChR: muscarinic acetylcholine receptor
MeCP2: methyl CpG-binding protein 2
miRNA: microRNA
PRh: perirhinal cortex
